# Auditory Stimuli Most Effective in Eliciting Reactivity in Critically Ill Patients with an Impaired Consciousness

**DOI:** 10.1007/s12028-026-02457-8

**Published:** 2026-02-11

**Authors:** Wolmet E. Haksteen, Lis N. K. Zandbergen, Nick Eleveld, Janneke Horn, A. Fleur van Rootselaar

**Affiliations:** 1 Department of Intensive Care, Amsterdam Neuroscience, Amsterdam UMC, University of Amsterdam, Meibergdreef 9, 1105 AZ Amsterdam, The Netherlands; 2 Department of Neurology and Clinical Neurophysiology, Amsterdam Neuroscience, Amsterdam UMC, University of Amsterdam, Meibergdreef 9, 1105 AZ Amsterdam, The Netherlands

**Keywords:** Critical care, Brain injuries, Electroencephalography, Coma, Reactivity

## Abstract

**Background:**

Electroencephalograpy (EEG) reactivity may aid functional outcome prediction in the intensive care unit (ICU). Different types of stimuli could vary in their ability to evoke reactivity. This study explored which type of stimulus is best to elicit EEG reactivity in ICU patients with an impaired consciousness. We also investigated associations between reactivity and hospital survival and functional outcome. Lastly, we compared standardized testing with random stimuli application.

**Methods:**

Adult ICU patients with an impaired consciousness and an EEG with reactivity testing were screened for eligibility. Stimuli were auditory (clapping and calling patient’s name), visual, tactile, and noxious. Three raters, blinded to clinical outcomes, scored each EEG recording and stimulus for reactivity. Interrater reliability was assessed using Fleiss’ kappa. Associations between reactivity and outcomes were analyzed with logistic regression.

**Results:**

In total, 72 patients were included, most commonly with traumatic brain injury which was present in 19 patients (26.4%) and a median GCS score of 6 (interquartile range, IQR 3–11). EEG reactivity was present in 30.6% of patients. Auditory stimuli were most effective, accounting for 57% of all reactive stimuli. Interrater reliability was highest for clapping (*κ* = 0.633, 77% raw agreement) and calling the patient’s name (*κ* = 0.584, 74% raw agreement). Reactivity was not associated with hospital survival or 6-month functional outcome. Standardized testing was applied in 23.6% of patients and improved raters’ certainty in detecting reactivity compared with the application of random stimuli (88.2% vs. 41.8%, p = 0.002).

**Conclusions:**

Auditory stimuli (clapping and calling the patient’s name) proved most effective in eliciting EEG reactivity. Reactivity was not associated with outcomes. Standardized testing increased raters’ certainty in detecting reactivity.

**Supplementary Information:**

The online version contains supplementary material available at 10.1007/s12028-026-02457-8.

## Introduction

Functional outcome prediction is an important aspect of intensive care unit (ICU) care, especially for patients with an impaired consciousness following acute brain injury, cardiac arrest, or systemic illness [1–3]. Electroencephalograpy (EEG) has emerged as a valuable tool in this context owing to its non-invasive nature and ability to be performed at the bedside, making it particularly suitable for critically ill patients [4]. Current international guidelines for neuroprognostication include recommendations for the use of EEG recordings in patients with an impaired consciousness [2, 5–8]. Several of these guidelines address EEG reactivity as a potential marker for outcome prediction [5, 7, 8]. EEG reactivity, a change in amplitude and/or frequency of the EEG following external stimuli, appears to be associated with better neurological outcomes [9–14]. A systematic review by Azabou et al. [15] that included a total of 42 articles concluded that it is a valuable tool for outcome prediction in patients with an impaired consciousness in the ICU, although methods of EEG reactivity testing may be heterogeneous.

This heterogeneity refers to differences in the number and type of stimuli across studies, ranging from only auditory stimuli to combinations of noxious, auditory, visual and tactile stimuli. Noxious stimuli appear to provoke reactivity most reliably but may not always be performed owing to ethical objections or a primary injury that prevents the use of supraorbital pressure or sternal rub [16–18]. Auditory stimuli show varying effectiveness in provoking EEG reactivity, possibly because the application of auditory stimuli may differ greatly between administrators in voice volume and strength [19–21]. Other commonly used stimuli include passive eye opening and tactile stimuli.

Standardized reactivity testing protocols that combine different types of stimuli could potentially improve the reliability of EEG reactivity for neuroprognostication as repeating the same or different stimuli could uncover subtle reactivity or confirm its absence [18]. However, extensive testing protocols inevitably increase bedside and visual assessment time. Identifying which stimulus types truly add diagnostic value is therefore essential to develop a practical and evidence-based standardized stimulation protocol. This could provide clinicians with information regarding the functional outcome and aid in decisions concerning the continuation or withdrawal of life-sustaining therapies in the ICU.

In this study, we investigated whether specific types of stimuli, e.g., auditory, visual, tactile and noxious, are more likely to induce EEG reactivity in patients with an impaired consciousness in the ICU. We also studied the association between EEG reactivity and hospital mortality and functional outcome within 6 months. Additionally, we explored the use of a standardized EEG reactivity testing protocol compared with the application of random stimuli. We hypothesized that auditory and noxious stimuli would be most effective in eliciting EEG reactivity and that reactivity is associated with better functional outcomes and survival. We also hypothesized that the use of a standardized EEG reactivity testing protocol increases the likelihood of detecting reactivity.

## Methods

### Design

This was a single-center observational study performed at the Amsterdam University Medical Center. Eligible patients who were admitted to the ICU between January 1, 2023 and January 1, 2025 were included. The cohort consisted of both prospectively included patients (IMPROVE-DOC study) and retrospectively included patients (REACT-DOC study). The local Medical Ethical Committee approved the IMPROVE-DOC study on December 5, 2022 (reference number: 2022–0661–NL82013.018.22) and the REACT-DOC study on May 21, 2025 (reference number: 2025.0026). Deferred consent was obtained from legal representatives of all prospectively included patients within 72 h after enrollment. Whenever possible, consent was also obtained from the patients themselves if they had regained consciousness at the 6-month follow-up telephone conference. For retrospectively included patients, a no-objection procedure was used: patients were informed of the intention to include them in the study and were given the opportunity to decline participation within four weeks of notification. For deceased patients, the need for a no-objection procedure was waived.

### Study Population

Patients were eligible for the current study if the following inclusion criteria were met: (1.) adult (≥ 18 years old) patients with an impaired consciousness and inability to follow commands (GCS motor score < 6) and (2.) a routine clinical EEG (minimum duration 30–60 min) with reactivity testing during ICU stay. For context, the IMPROVE-DOC study is a large observational study which focuses on more modalities than just EEG recordings. Therefore, patients in this cohort were subject to additional inclusion criteria. If patients were previously not eligible for the IMPROVE-DOC study, they were retrospectively included in the REACT-DOC study if the two inclusion criteria of the current study were met.

The IMPROVE-DOC population consisted of patients admitted to the ICU with severe acute brain injury defined as traumatic brain injury, subarachnoid hemorrhage, intracerebral hemorrhage, ischemic stroke, or meningo-encephalitis. The Glasgow Coma Scale (GCS) total score should not have exceeded eight prior to inclusion, which took place within 24 h after sustaining brain injury. Patients with a previously diagnosed neurodegenerative disease or life-expectancy ≤ 6 months due to another disease were excluded. Patients included retrospectively through the REACT-DOC study were admitted with diagnoses including severe infections or sepsis, complications following cardiac surgery, metabolic derangements, and epilepsy. A subset of these patients presented with severe acute brain injury, similar to the patients in the IMPROVE-DOC study. Exclusion criteria encompassed patients who underwent EEG as a confirmatory test for the determination of brain death in the context of organ donation, as well as those receiving continuous EEG monitoring for neuroprognostication following out of hospital cardiac arrest. Patients were identified using a departmental log of all EEGs performed in the ICU.

### Patient Characteristics

Demographic, clinical and outcome data were collected from the electronic patient records. Demographic characteristics included: age, sex, ICU admission diagnosis, GCS score and pupillary reactivity at admission and the Clinical Frailty Scale (CFS). The CFS was dichotomized into “frail” (score of ≥ 5) and “non-frail” (score of ≤ 4) [22]. The highest Sequential Organ Failure Assessment (SOFA) score during ICU admission was documented.

### EEG Baseline Characteristics and Reactivity Testing

EEGs were recorded with a BrainRT EEG system with SD Plus amplifier (Micromed, OSG, Kontich, Belgium), with 1024 Hz sampling frequency. A minimum of 9 electrodes were used following the international 10 to 20 system. The indication and timing of the EEG (in days after ICU admission) were documented. Additionally, the GCS score in the hour prior to the EEG recording and the level of sedation during the EEG were described. Sedation levels during the EEG were categorized according to the classification proposed by Amiri et al. [23] into: “none or minimal,” “low to moderate,” “high or very high,” and “unknown.” The potential residual effects of long-acting sedative agents were taken into account when determining the patient’s sedation level at the time of the EEG recording. Reactivity testing was performed by trained neurophysiology technicians and trained members of the IMPROVE-DOC research team. Patients who were prospectively included in the IMPROVE-DOC study followed a standardized EEG reactivity testing protocol, consisting of: auditory stimuli (clapping and calling the patient’s first name), visual stimuli (passive eye opening), tactile stimuli (nasal tickle using a cotton swab) and noxious stimuli (sternal rub) (Supplementary Fig. S1). Each stimulus was applied for a duration of 5 s, and an interval of 30 s was applied between stimuli. The entire set of five stimuli was repeated thrice. If patients physically responded to a noxious stimulus (e.g., opened their eyes), this stimulus was not repeated. The standardized protocol was implemented as part of routine clinical practice at our hospital since October 2023. Therefore, several retrospectively included patients also underwent the standardized protocol. For the remaining retrospectively included patients, one or more of the aforementioned stimuli were applied at least twice to assess reactivity. Noxious stimuli in this group could consist of either a sternal rub, peripheral stimulation or orbital pressure.

### ***EEG (Pre)processing***

EEGs were reviewed prior to analyses using BrainRT software (RT Software Suite Version 4.03.00 Build 5049) with video recordings to correct stimulus annotations if needed (LZ). The recordings were exported (EDF +) and preprocessed using MATLAB (version R2023b) with the FieldTrip toolbox (version 20241025). Preprocessing steps included bandpass filtering (0.3–70 Hz), resampling to 256 Hz and segmentation around annotated stimuli. Segments of 2 min prior to the start of the first stimulus and 2 min after the last stimulus were selected to assess the background pattern. For assessment of reactivity per stimulus, epochs of 30 s were selected, consisting of 15 s prior to and 15 s after the stimulus. EEG recordings severely obscured by eye movements, muscle artifacts and electrical noise were excluded from analysis.

### EEG Background and Reactivity Scoring

A custom-made interactive MATLAB graphical user interface (GUI) was developed for EEG scoring. The GUI displayed a nine-electrode reduced montage (Supplementary Fig. S2). Three independent raters (WH, FvR, JH) blinded to clinical patient characteristics and outcomes scored all EEGs.

For EEG background scoring, EEGs were randomly assorted. The background pattern was categorized into: “(nearly) continuous” (< 10% of recording suppressed or attenuated), “discontinuous” (10–49% of recording suppressed or attenuated), “burst suppression” (50–99% of recording suppressed or attenuated), or “suppressed” (> 99% of recording suppressed or attenuated) [24]. Additionally, the dominant frequency (> 8 Hz or < 8 Hz) and the presence of rhythmic and/or periodic patterns (periodic discharges, rhythmic delta, or spike-wave) were documented [24].

For EEG reactivity scoring, EEGs were again randomly assorted, but all stimulus-containing epochs from a given patient were scored consecutively before moving on to the next EEG. Reactivity was defined as any change in frequency and/or amplitude of the EEG within 15 s following a stimulus. Each stimulus was assessed individually and could be scored as either “reactive” or “unreactive.” Raters additionally indicated their certainty regarding the presence of reactivity and whether artifacts may have interfered with EEG interpretation.

### Outcome Measures

Hospital mortality and the Glasgow Outcome Scale Extended (GOSE) score within 6 months were primary outcome measures [25]. The GOSE score was dichotomized; a score of ≥ 5 was considered a favorable outcome. For the patients who were included prospectively in the study, follow-up took place at 6 months using a conference call with the patient and/or the legal representative. For patients who were retrospectively included in the study, the GOSE score was inferred from the data available in the clinical notes within 6 months after hospital admission. The score found closest to the 6-month follow-up timepoint was used. Other outcome variables were the length of ICU and hospital stay.

### Statistical Analysis

Continuous data were presented as medians and interquartile ranges (IQR) or means and standard deviations (SD) depending on statistical distribution, which was tested using histograms, Q–Q plots and the Shapiro–Wilk Test. Categorical data were presented as frequencies and percentages. Missing data were assessed per variable and reported when present.

First, we determined stimulus effectiveness to assess if certain types of stimuli were more likely to elicit reactivity. Each individual stimulus was determined reactive if at least two out of three raters scored this stimulus as reactive. For each stimulus type (clapping, calling name, passive eye opening, nasal tickle, noxious), stimulus effectiveness was calculated as the proportion of reactive stimuli relative to the total number of applied stimuli of that stimulus type. Inter-rater reliability was assessed per stimulus type using Fleiss’ kappa, which is appropriate for multiple raters and accounts for agreement occurring by chance. A threshold > 0.4 indicates moderate interrater agreement, a threshold > 0.6 indicates substantial interrater agreement, and a threshold of > 0.8 indicates almost perfect interrater agreement [26]. Percent agreement values were also reported to provide the raw proportion of stimuli raters fully agreed on. Additionally, an alluvial plot was created to visualize how each individual reactive stimulus, within its stimulus type, relates to the dichotomized GOSE score within 6 months.

Second, univariable logistic regression was used to analyze the association between presence of overall EEG reactivity and the outcome measures hospital survival and the GOSE score within 6 months. The EEG recording was scored overall reactive if two or more stimuli, regardless of whether they were of the same stimulus type, were determined reactive by majority vote. Additionally, age and GCS at admission were included in a multivariable logistic regression model as known prognostic variables, sedation level was included as well to account for its potential influence on the occurrence of reactivity. Odds ratios (OR) and corresponding *p*-values were reported.

Last, we assessed differences in baseline variables and EEG findings between patients who underwent the standardized EEG reactivity testing protocol and patients who received only random stimuli using the Mann–Whitney *U* test or Student’s *t*-test for continuous variables and the Fisher’s exact test and chi-squared test for categorical variables. All statistical analyses were performed using R (version 4.2.3). A *p*-value of < 0.05 was considered statistically significant.

## Results

In a total of 72 patients, an EEG with reactivity testing was conducted (Supplementary Fig. S3). The most frequent admission diagnosis was traumatic brain injury (26.4%) followed by an impaired consciousness after cardiac surgery/cardiac event and severe infection/sepsis (Table 1). The median GCS score at ICU admission was 6 (IQR 3–11), and the highest median SOFA score during ICU admission was 10 (IQR 7–12). The median duration of ICU admission was 12 days (IQR 6–26) and the median hospital stay was 29 days (IQR 16–57). Of the 34 deceased patients, 26 died following withdrawal of life-sustaining therapy (WLST). A total of 38 patients (52.8%) were alive at hospital discharge. In 14 patients (19.4%), a favorable GOSE score could be identified within 6 months.

**Table 1:** Baseline characteristics

	***n* = 72**
*Baseline characteristics*	
Age, years	59 (15.5)
Female, *n* (%)	31 (43.1)
*Admission diagnosis, n (%)* Traumatic brain injury Cardiac surgery/cardiac event Severe infection/sepsis Meningo-encephalitis Subarachnoid hemorrhage Intracerebral hemorrhage Epilepsy Metabolic derangements Ischemic stroke Other^(1)^	19 (26.4) 10 (13.9) 10 (13.9) 7 (9.7) 6 (8.3) 5 (6.9) 5 (6.9) 4 (5.6) 2 (2.8) 4 (5.6)
*Relevant medical history, n (%)*^*(2)*^ Cardiovascular Diabetes Pulmonary Neurological disorders Psychiatric disorders Unknown	34 (47.2) 19 (26.4) 18 (25.0) 16 (22.2) 4 (5.6) 2 (2.8)
Pupillary reactivity = two pupils reactive, *n* (%)	59 (81.9)
*Clinical Frailty Scale score, n (%)*^*(3)*^ Frail Non-frail	7 (9.7) 65 (90.3)
GCS score at admission	6.00 [3–11]
*EEG baseline characteristics*	
Timing of EEG after ICU admission, days	4 [1–10]
GCS total score during EEG	6 [4–7]
*Sedative medication during EEG, n (%)* None or minimal Low or moderate High or very high Unknown	55 (76.4) 10 (13.9) 6 (8.3) 1 (1.4)

Values are presented as mean and (standard deviation), median and [interquartile range], or counts and (percentages)

^(1)^Three patients presented with brain metastases and one patient was admitted with a severe distributive shock

^(2)^% do not add up to 100% as for some patients multiple medical history categories are applicable

^(3)^“Frail” is defined as a Clinical Frailty Scale score ≥ 5; “Non-frail” is defined as a Clinical Frailty Scale score ≤ 4

*EEG* electroencephalography, *GCS* Glasgow Coma Scale score, *ICU* intensive care unit

The EEG recordings took place after a median of 4 days following ICU admission (IQR 1–10) (Table 1). The main reason for requesting an EEG was to rule out (non-convulsive) status epilepticus ((NC)SE). A continuous background pattern was most common and found in 67 patients (93.1%); the background frequency was > 8 Hz in only 21 patients (29.2%) (Table 2). Epileptiform activity was detected in eight patients (11.1%); (NC)SE was diagnosed in four patients (5.6%). EEG reactivity was present in 22 patients (30.6%).

**Table 2:** EEG findings

	***n* = 72**
*Background pattern, n (%)* Discontinuous Continuous N/A	2 (2.8) 67 (93.1) 3 (4.2)
*Background frequency, n (%)* < 8 Hz > 8 Hz N/A	46 (63.9) 21 (29.2) 5 (6.9)
Epileptiform activity, *n* (%)^(1)^	8 (11.1)
Number of stimuli applied per patient	5 [3–8]
Number of reactive stimuli per patient	1 [0–2]
*Reactivity overall, n (%)*^*(2)*^ Present Absent	22 (30.6) 50 (69.4)

Values are presented as mean and (standard deviation), median and [interquartile range], or counts and (percentages)

^(1)^Epileptiform activity is defined as the presence of rhythmic an/or periodic patterns. detection of epileptiform activity is based on the rating of FR, our most experienced rater (neurologist and clinical neurophysiologist)

^(2)^Overall reactivity was scored present if at least two stimuli were determined reactive by majority vote

*EEG* electroencephalography

**Fig. 1:**
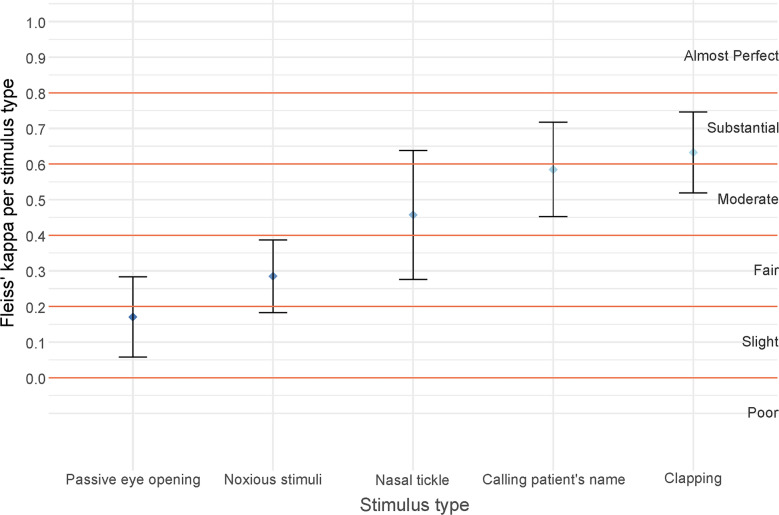
Visualization of the Fleiss’ kappa values per stimulus type; exact values can be found in Table 3

**Fig. 2:**
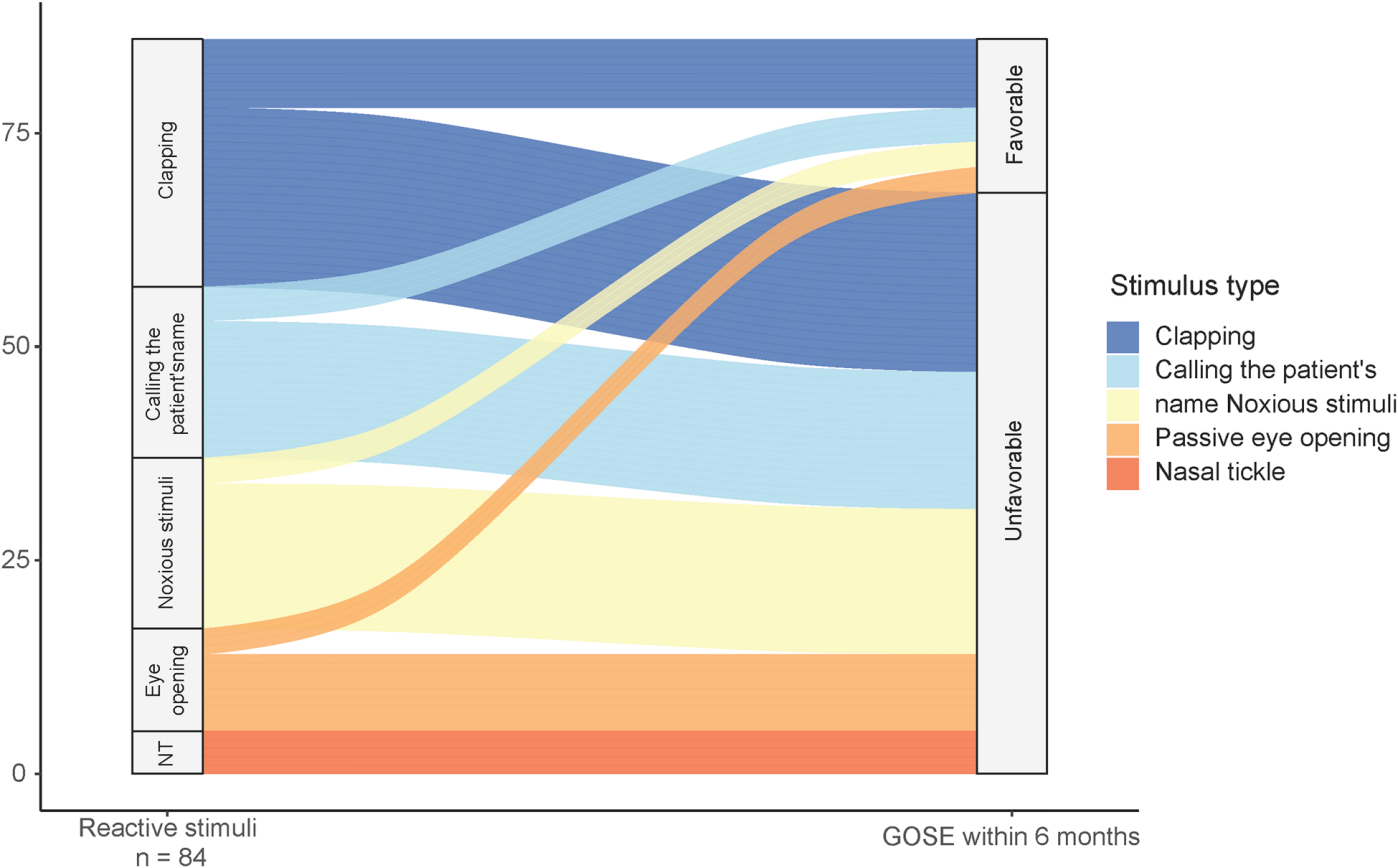
Alluvial plot depicting how each individual reactive stimulus, within its stimulus type, relates to the 6-month dichotomized GOSE score. The first column represents all reactive stimuli (*n* = 84) categorized into the stimulus types (clapping, calling the patient’s name, passive eye opening, nasal tickle, and noxious stimuli). A stimulus was determined reactive if at least two out of three raters scored it as such. The second column shows the transition to a “favorable” (GOSE ≥ 5) or “unfavorable” (GOSE ≤ 4) functional outcome within 6 months. *GOSE* Glasgow Outcome Scale Extended score, *NT* nasal tickle

In total, 437 stimuli were applied (Table 3). Overall, 86 stimuli were scored as reactive, resulting in a stimulus effectiveness of 19.7%. The percentage of stimuli that the raters interpreted the exact same way was 71.4%, and the Fleiss’ kappa was 0.449, indicating moderate agreement. When looking at individual stimulus types, auditory stimuli (clapping and calling the patient’s name) were the most effective in eliciting EEG reactivity (29% and 27.4%). Together, these auditory stimuli accounted for 57% of all reactive stimuli. The auditory stimuli types also reached the highest Fleiss’ kappa and percent interrater agreement values, respectively, a Fleiss’ kappa value of 0.633 and 77% agreement for clapping and a Fleiss’ kappa value of 0.584 and 74% agreement for calling the patient’s name (Fig. 1). Only 16.3% of the noxious stimuli were reactive with a Fleiss’ kappa value of 0.285 indicating fair agreement and percent interrater agreement of 66.7%. Of all reactive stimuli, clapping was most often observed to relate to a favorable outcome, followed by calling the patient’s name and noxious stimuli. The stimulus-type nasal tickle never led to a favorable outcome when scored reactive. In seven patients (9.7%), the raters identified artifacts in the EEG recordings that could have affected their assessment of reactivity (Fig. 2).

**Table 3:** Stimulus effectiveness and inter-rater reliability

Per stimulus type	Number applied^(1)^	Number reactive^(2)^	Percent reactive^(3)^	Fleiss’ kappa^(4)^	Percent agreement^(5)^
Clapping	100	29	29.0	0.633	77.0
Calling name	73	20	27.4	0.584	74.0
Passive eye opening	102	12	11.8	0.170	67.6
Nasal tickle	39	5	12.8	0.457	76.9
Noxious stimulus	123	20	16.3	0.285	66.7
**Total**	437	86	19.7	0.449	71.4

^(1)^The total number of stimuli applied across all patients

^(2)^The total number of reactive stimuli across all patients, a stimulus was determined reactive if the majority of the raters scored this stimulus as reactive

^(3)^Effectiveness of the stimulus in percentages calculated by dividing the total number of reactive stimuli by the total number of applied stimuli

^(4)^Fleiss’ kappa reports the agreement between raters, correcting for chance

^(5)^Percent agreement provides the raw proportion of stimuli raters fully agreed on, without adjusting for chance

*EEG* electroencephalography

**Fig. 3:**
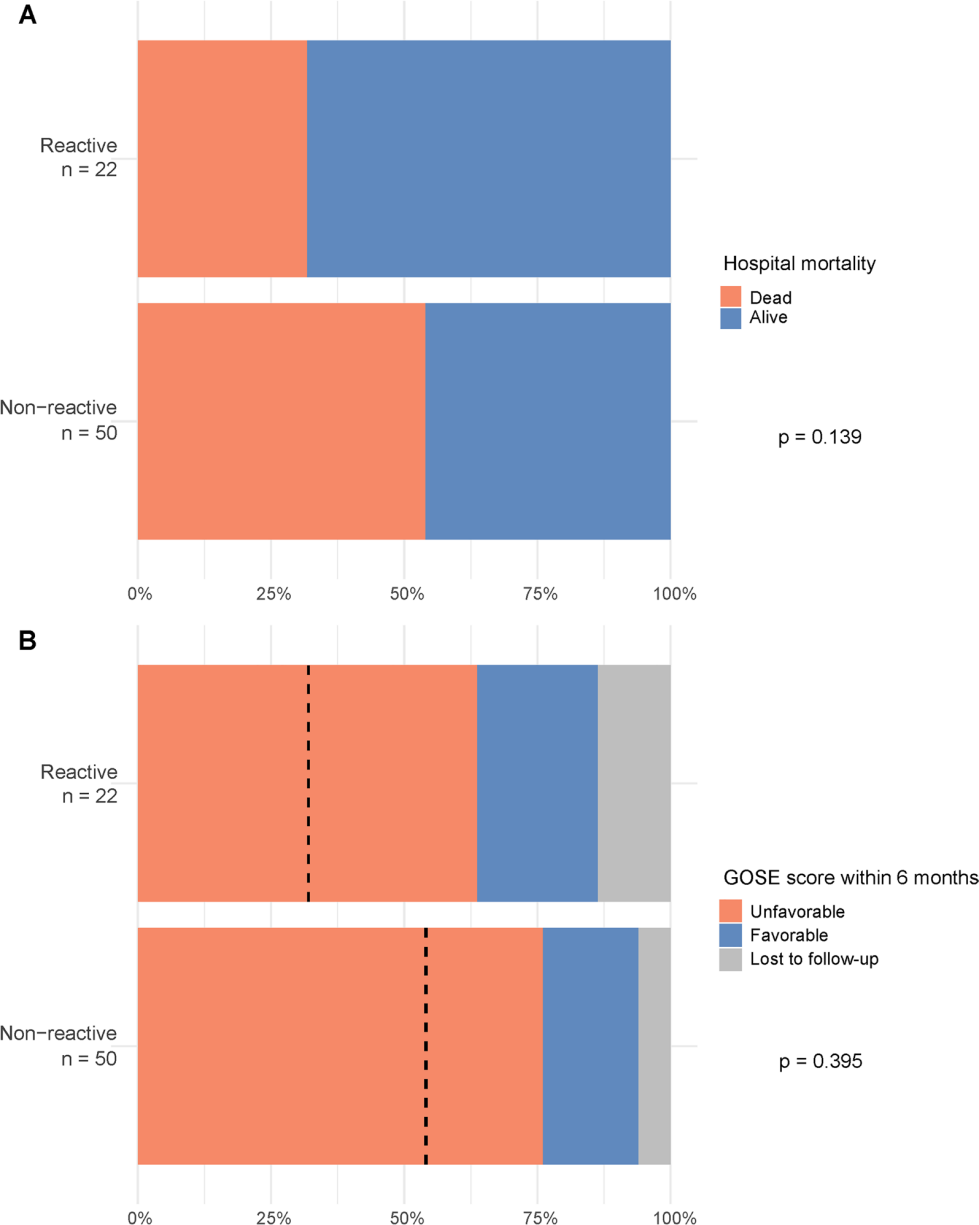
Bar charts, representing in **A**: proportion of hospital survivors and non-survivors among patients with a reactive EEG versus a non-reactive EEG. An EEG recording was scored overall reactive if at least two stimuli were determined reactive by the majority vote of three raters. In **B**, the distribution of the dichotomized GOSE scores within 6 months in patients with a reactive versus a non-reactive EEG. The dashed lines indicate hospital mortality (represented in **A**). A favorable outcome is defined as a GOSE score ≥ 5. *p*-Values were derived using the chi-squared test. *EEG* electroencephalography, *GOSE* Glasgow Outcome Scale Extended scale

Survival at hospital discharge was 68.2% in patients with a reactive EEG compared with 46% in patients with a non-reactive EEG, but this was not a statistically significant difference (*p* = 0.139) (Fig. 3A). An association between hospital survival and presence of reactivity could not be identified in both univariable (OR 2.51, 95% confidence intervals (95%CI) 0.89–7.58, *p* = 0.09) and multivariable (OR 3.35, 95%CI 0.95–13.5, *p* = 0.07) models. Similarly, we were unable to identify a difference in 6-month GOSE scores between patients with a reactive EEG and patients with a non-reactive EEG (*p* = 0.395) (Fig. 3B). The univariable (OR 1.42, 95%CI 0.39–4.81, *p* = 0.58) and multivariable (OR 2.07, 95%CI 0.35–12.1, *p* = 0.40) models also did not indicate an association between GOSE scores within 6 months and presence of reactivity. However, in the reactive EEG group, survival remained 68.2%, indicating no additional patients died following hospital discharge.

In 17 patients (23.6%), the standardized EEG reactivity testing protocol was applied (Supplementary Table S1). The EEG was determined overall reactive in 41.2% of patients in the standardized EEG reactivity testing protocol group compared with 27.3% of patients in the random stimuli group (*p* = 0.432) (Supplementary Table S2). Raters more often reported to be certain regarding the presence of reactivity in the EEG in the standardized protocol group (88.2%) compared with the random stimuli group (41.8%) (*p* = 0.002).

## Discussion

In this study, we found that auditory stimuli were most effective in eliciting EEG reactivity and that both clapping and calling the patient’s name showed substantial Fleiss’ kappa values and high inter-rater agreement percentages. An association between the presence of EEG reactivity and hospital survival or better GOSE scores within 6 months could not be identified. Furthermore, we found that the certainty of raters increased when reactivity was tested using a standardized protocol.

Both auditory and noxious stimuli have shown to be useful for eliciting EEG reactivity [18]. Interestingly, in our study population, only a small proportion of noxious stimuli evoked reactivity, even though these were applied a similar number of times as auditory stimuli. An explanation for this finding could be that the application of a sternal rub may cause (muscle) artifacts which interfere with the interpretation of reactivity [20]. In our study population, nearly 10% of the EEG recordings contained artifacts that may have obscured reactivity assessment. However, as the presence of artifacts was scored for the entire EEG recording and not per stimulus, their effect on the assessment of noxious stimuli remains uncertain. Additionally, the variation in application force of noxious stimuli between administrators could have played a role in the low effectiveness of noxious stimuli. Another explanation could be the use of sedative medication as in approximately a quarter of the patients the sedation levels were “low or moderate” to “high or very high” [27]. This may also offer insight into why in nearly 65% of the patients a slowed background pattern (< 8 Hz) was observed. Nevertheless, there are many examples of studies in postanoxic patients where sedative medication was continued during the EEG recording and EEG reactivity could still be detected [19, 28, 29].

In our study population, we were unable to identify an association between better functional outcomes and the presence of reactivity. This finding is surprising as many studies have indicated the prognostic value of EEG reactivity in various types of patient populations [15]. Bouchereau et al. [30] identified a quantitative EEG reactivity marker that was independently associated with awakening and 3-month consciousness levels in patients with severe brain injury. However, in their study population, almost 75% of the patients regained consciousness in the ICU, and in more than 70%, EEG reactivity was observed. In our study population, only half survived hospital admission and EEG reactivity could be identified in less than a third which likely entails that we included more severely ill patients. This is also reflected in the low GCS at ICU admission and high SOFA score. A study conducted by Egbekibe et al. [31] included patients with a more similar severity profile. They had a low admission GCS score, and a favorable 6-month GOSE score could be identified in only 11% of the patients. In this study, the presence of cognitive–motor dissociation, brain activation in the absence of behavioral response to spoken commands, was assessed in EEGs using a machine learning algorithm. The presence of cognitive–motor dissociation was associated with better neurological outcome, and differences between patients with and without cognitive–motor dissociation were evident as early as 3 months post-injury. A difference with this study was the sample size. They included over 190 patients, suggesting our cohort may have been too small to identify significant differences. Nonetheless, in our results the odds ratios indicated positive trends for the relation between EEG reactivity and 6-month GOSE score. For reactivity and hospital survival, we found borderline significant results. Another interesting finding was that all patients discharged with a reactive EEG were still alive at the 6-month follow-up, whereas in the non-reactive group, mortality increased. Overall, these findings suggest that EEG reactivity is likely an important marker of outcome, and we hypothesize that, in a larger or better-powered study, this association may have been confirmed.

We only investigated the use of a standardized EEG reactivity testing protocol to a limited extent as baseline characteristics differed significantly between the standardized testing group and random stimuli group which may have influenced the occurrence of reactivity. These differences likely reflect the distinct inclusion methods, with most standardized testing protocol patients enrolled via the IMPROVE-DOC study. Owing to the small sample size and these differences at baseline, considerable confounding must be assumed and analyses to assess the association between protocol type and clinical outcomes (hospital mortality, GOSE score at 6 months) were not performed. However, an important observation was that raters expressed greater certainty in their assessment of EEG reactivity when the standardized protocol was used. This certainty likely stems from the use of multiple stimulus types and the repetition of stimuli, allowing reactivity to a given stimulus to be confirmed through reproducibility. Similarly, Caroyer et al. [32] assessed the use of a standardized EEG reactivity protocol in post-anoxic patients and found that a combination of auditory and noxious stimuli led to the highest sensitivity for a favorable outcome. Our findings support the recommendation to implement a standardized EEG reactivity testing protocol [15, 18, 33]. On the basis of the results of this study, auditory and noxious stimuli appear to be most effective in eliciting reactivity. However, given that the presence of EEG reactivity may influence decisions on withdrawal of life-sustaining therapy, it must be assessed comprehensively. If there are no time-based or logistical constraints, the use of multiple types of stimuli (auditory, visual, tactile, and noxious) should be considered. Additionally, the use of a standardized protocol with repeating stimuli, may increase raters’ certainty in detecting EEG reactivity. Future studies should aim to systematically develop, implement and evaluate such standardized protocols. Moreover, emerging quantitative methods for EEG reactivity detection may ultimately provide a more objective alternative to visual EEG reactivity assessment. Recent findings, including those of Bouchereau et al., show promising results in this field but require validation in larger cohorts [30]. Until validated quantitative markers are established, visual assessment by experienced raters remains a reliable option.

### Strengths and Limitations

This study was conducted in a large neurosurgical ICU, which improves the credibility and generalizability of the results. Another strength is that EEG reactivity was assessed by three independent raters, blinded to clinical outcomes. A limitation of the study is the modest sample size and (partially) retrospective design. The selection of patients using two different inclusion methods led to considerable variability in patients and the reactivity testing protocols applied. This limited extensive statistical analysis comparing the use of a standardized reactivity testing protocol versus random stimuli application.

## Conclusions

In ICU patients, auditory stimuli (clapping and calling the patient’s name) proved most effective in eliciting EEG reactivity. No significant association was found between EEG reactivity and hospital survival and functional outcome within 6 months, possibly due to the small sample size and severely ill patient population. Standardized EEG reactivity testing increased raters’ certainty in detecting reactivity.

## Supplementary Information

Below is the link to the electronic supplementary material.

Supplementary file1 (PDF 302 KB)
