# Supplementary material for: Auditory Stimuli Most Effective in Eliciting Reactivity in Critically Ill Patients with an Impaired Consciousness
Source: Neurocrit Care. 2026 Feb 11;45(1):171–80. doi: 10.1007/s12028-026-02457-8 (PMC13369764; doi:10.1007/s12028-026-02457-8)
Supplement: Supplementary file 1 — Supplementary file1 (PDF 302 KB) [file 12028_2026_2457_MOESM1_ESM.pdf]

Supplementary material – Neurocritical Care

Manuscript: 'Auditory stimuli most effective in eliciting reactivity in critically ill patients with an impaired consciousness'

Authors: Wolmet E. Haksteen[1*] MD, Lis N.K. Zandbergen[1*], Nick Eleveld[2], Janneke Horn[1] MD PhD, A. Fleur van Rootselaar[2] MD PhD

Affiliations:

1. Amsterdam UMC, University of Amsterdam, Department of Intensive Care, Amsterdam Neuroscience, Meibergdreef 9, Amsterdam, Netherlands

2. Amsterdam UMC, University of Amsterdam, Department of Neurology and Clinical Neurophysiology, Amsterdam Neuroscience, Meibergdreef 9, Amsterdam, Netherlands

* Joint first authors

Corresponding author:

Wolmet E. Haksteen

w.e.haksteen@amsterdamumc.nl

ORCID: 0000-0002-2897-7302

Figure S1. Schematic representation of standardized EEG reactivity testing protocol

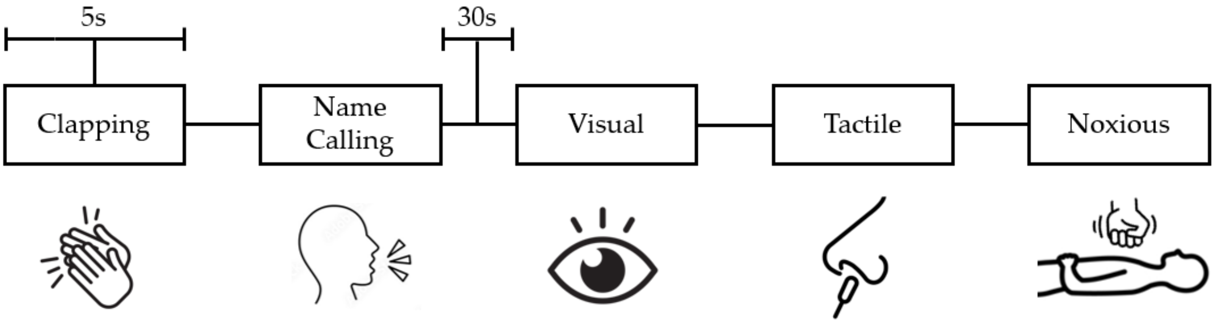

The standardized EEG reactivity testing protocol consisting of: auditory stimuli (clapping and calling the patient's first name), a visual stimulus (passive eye opening), a tactile stimulus (nasal tickle using a cotton swab) and a noxious stimulus (sternal rub). The entire set of 5 stimuli had to be repeated thrice.

Figure S2. Montage used for EEG scoring

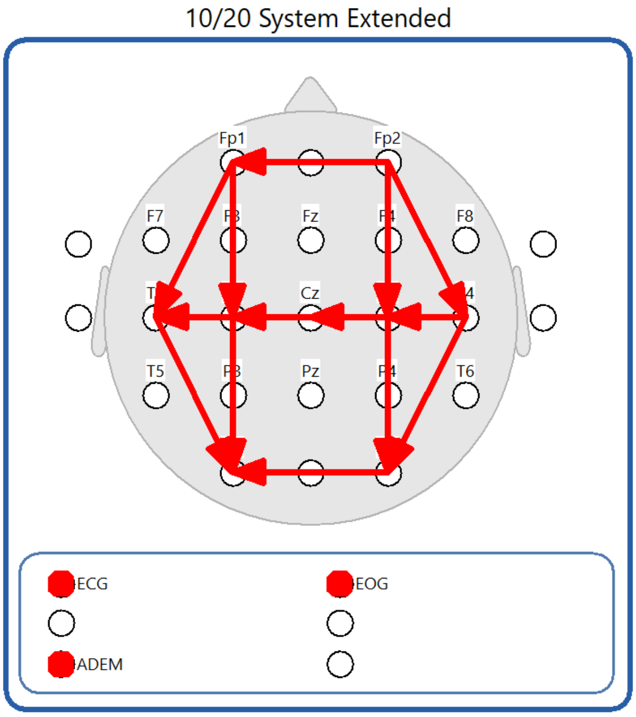

Reduced montage used for EEG scoring.
EEG = Electroencephalography

Figure S3. Flowchart of patient selection

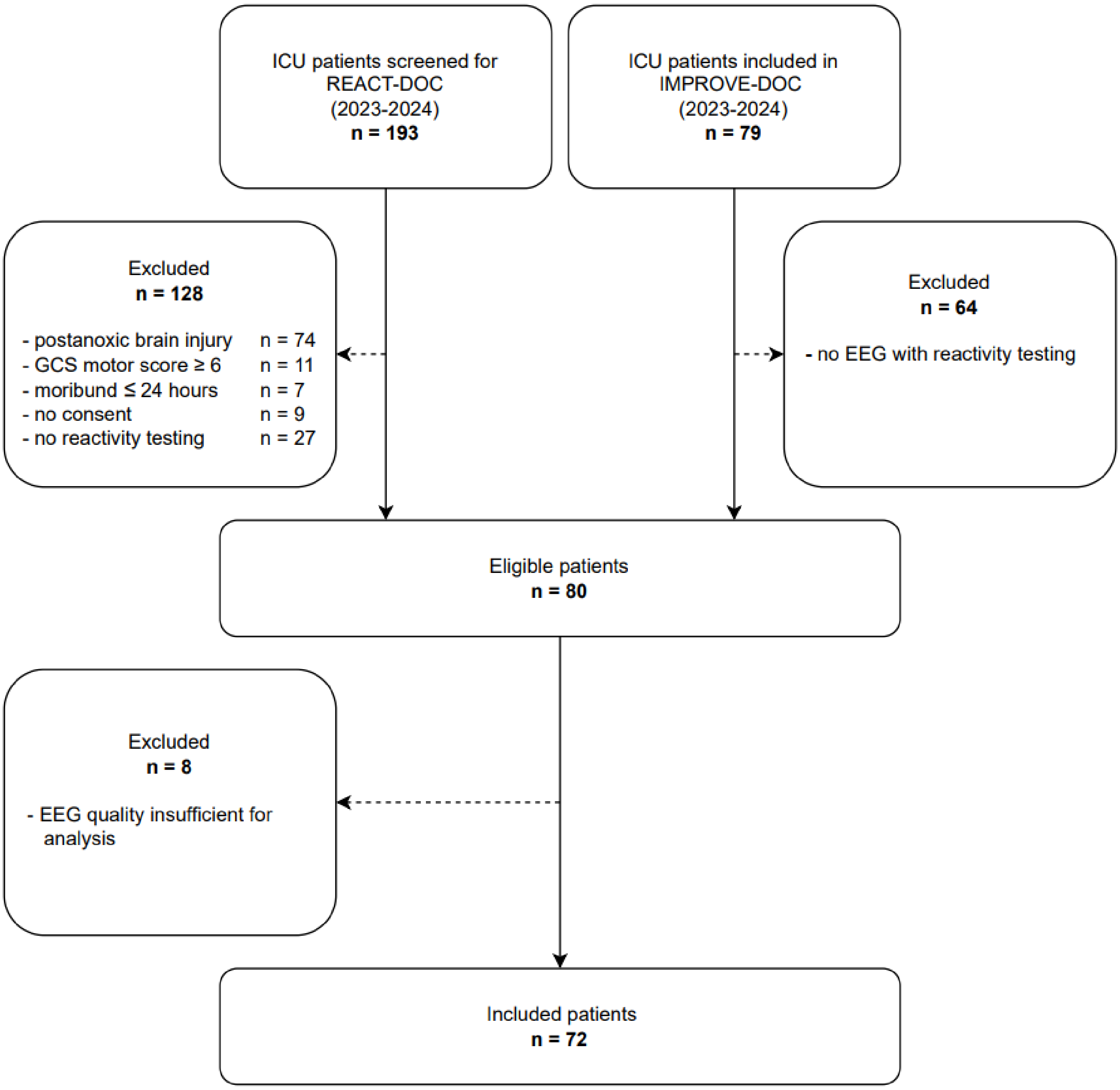

Flowchart depicting the patient selection process.
DOC = Disorders of Consciousness, EEG = electroencephalography, ICU = Intensive Care Unit

Table S1. Baseline characteristics stratified by completion of a standardized EEG reactivity testing protocol or application of random stimuli

	Standardized protocol n = 17	Random stimuli n = 55	p-value
Baseline characteristics			
**Study population, n (%)**			
IMPROVE-DOC	10 (58.8)	5 (9.1)	**<0.001**
REACT-DOC	7 (41.2)	50 (90.9)	
**Age, years**	48 (13.7)	62 (14.6)	**<0.001**
**Female, n (%)**	5 (29.4)	26 (47.3)	0.308
**Admission diagnosis, n (%)**			
Traumatic brain injury	9 (52.9)	10 (18.2)	**0.008**
Cardiac surgery/cardiac event	0	10 (18.2)	
Severe infection/sepsis	1 (5.9)	9 (16.4)	
Meningo-encephalitis	0	7 (12.7)	
Subarachnoid hemorrhage	2 (11.8)	4 (7.3)	
Intracerebral hemorrhage	2 (11.8)	3 (5.5)	
Epilepsy	1 (5.9)	4 (7.3)	
Metabolic derangements	0	4 (7.3)	
Ischemic stroke	2 (11.8)	0	
Other(1)	0	4 (7.3)	
**Pupillary reactivity = two pupils reactive, n (%)**	9 (52.9)	50 (90.9)	**0.004**
**Clinical Frailty Score, n (%)(2)**			
Frail	1 (5.9)	6 (10.9)	0.886
Non-frail	16 (94.1)	49 (89.1)	
**GCS score at admission**	4.5 [3-6]	9 [3-13]	**0.012**
EEG baseline characteristics			
**Timing of EEG after ICU admission, days**	7 [1-13]	4 [1-9]	0.346
**GCS total score during EEG**	5 [4-7]	6 [4-7]	0.358
**Sedative medication during EEG, n (%)**			
None or minimal	15 (88.2)	40 (72.7)	0.248
Low or moderate	0	10 (18.2)	
High or very high	2 (11.8)	4 (7.3)	
Unknown	0	1 (1.8)	

Values are presented as mean and (standard deviation), median and [Interquartile range], or counts and (percentages).
(1) Three patients presented with brain metastases and one patient was admitted with a severe distributive shock.
(2) 'Frail' is defined as a Clinical Frailty Scale score ≥ 5, 'Non-frail' is defined as a Clinical Frailty Scale score ≤ 4.
EEG = Electroencephalography, GCS = Glasgow Coma Scale score, ICU = Intensive Care Unit

Table S2. EEG findings stratified by completion of a standardized stimuli protocol or application of random stimuli

	Standardized testing protocol (n = 17)	Random stimuli (n = 55)	p-value
**Background Pattern, n (%)**			
Discontinuous	1 (5.9)	1 (1.8)	0.427
Continuous	16 (94.1)	51 (92.7)	
N/A	0	3 (5.5)	
**Background Frequency, n (%)**			
<8 Hz	10 (58.8)	36 (65.5)	0.814
>8 Hz	6 (35.3)	15 (27.3)	
N/A	1 (5.9)	4 (7.3)	
**Epileptiform activity**, **n (%)[(1)]**	2 (11.8)	6 (10.9)	1.000
**Number of stimuli applied per patient**	14 [12-15]	4 [3-5]	**<0.001**
**Number of reactive stimuli per patient**	1 [0-4]	1 [0-2]	0.234
**Reactivity overall, n (%)[(2)]**			
Present	7 (41.2)	15 (27.3)	0.432
Absent	10 (58.8)	40 (72.7)	

Values are presented as mean and (standard deviation), median and [Interquartile range], or counts and (percentages).
(1) Epileptiform activity is defined as the presence of rhythmic an/or periodic patterns. Detection of epileptiform activity is based on the rating of FR, our most experienced rater (neurologist and clinical neurophysiologist).
(2) Overall reactivity was scored present if at least two stimuli were determined reactive by majority vote.
EEG = Electroencephalography
